# Interventions for physical activity promotion applied to the primary healthcare settings for people living in regions of low socioeconomic level: study protocol for a non-randomized controlled trial

**DOI:** 10.1186/2049-3258-72-8

**Published:** 2014-03-13

**Authors:** Emanuel P Salvador, Evelyn H Ribeiro, Leandro MT Garcia, Douglas R Andrade, Vanessa MV Guimaraes, Marcelo S Aoki, Alex A Florindo

**Affiliations:** 1 Health Sicence Department, Cruzeiro do Sul University, Sao Paulo, Brazil; 2 School of Arts, Science and Humanity, University of Sao Paulo, Sao Paulo, Brazil; 3 School of Public Health, University of Sao Paulo, Sao Paulo, Brazil

**Keywords:** Health promotion, Intervention study, Public health practice, Physical activity

## Abstract

**Background:**

Regular physical activity practice has been widely recommended for promoting health, but the physical activity levels remain low in the population. Therefore, the study of interventions to promote physical activity is essential. Objective: To present the methodology of two physical activity interventions from the “Ambiente Ativo” (“Active Environment”) project.

**Methods:**

12-month non-randomized controlled intervention trial. 157 healthy and physically inactive individuals were selected: health education (n = 54) supervised exercise (n = 54) and control (n = 49). Intervention based on health education: a multidisciplinary team of health professionals organized the intervention in group discussions, phone calls, SMS and educational material. Intervention based on supervised exercise program: consisted of offering an exercise program in groups supervised by physical education professionals involving strength, endurance and flexibility exercises. The physical activity level was assessed by the International Physical Activity Questionnaire (long version), physical activities recalls, pedometers and accelerometers over a seven-day period.

**Result:**

This study described two different proposals for promoting physical activity that were applied to adults attended through the public healthcare settings. The participants were living in a region of low socioeconomic level, while respecting the characteristics and organization of the system and its professionals, and also adapting the interventions to the realities of the individuals attended.

**Conclusion:**

Both interventions are applicable in regions of low socioeconomic level, while respecting the social and economic characteristics of each region.

**Trial registration:**

ClinicalTrials.gov NCT01852981

## Background

Regular practice of physical activity has been widely recommended for promoting health and improving quality of life and wellness [1-4]. However, the proportion of physically active individuals is low, particularly in relation to leisure-time activities [5,6]. In middle-income countries like Brazil, this topic is also a matter of concern. According to data from a national survey in 2009, the prevalence of adults who were not attaining the recommended levels for leisure-time physical activity reached 85.3% [5]. In regions of low socioeconomic level, this situation is also worrying. In a study conducted on a sample of people living in a region of low socioeconomic level in the city of São Paulo, 68.8% of the adults were not even doing 10 minutes of physical activities per week during their leisure time [7]. Furthermore, the promotion of physical activity is also key to fighting obesity and especially chronic diseases [8]. According to the Surveillance of the Brazilian Ministry of Health, the prevalence of overweight and obesity in adults has increased significantly in Brazil, from 43% of overweight and 11.4% obesity in 2006 [9] to 48.5% and 15.8%, respectively, in 2011 [10]. Therefore, it is important to devise strategies aimed at increasing the population-level physical activity.

Several studies have evaluated new proposals for promoting physical activity using supervised exercise programs (broadly consisting of aerobic, anaerobic, stretching and game programs) and interventions based on health education [11,12]. Health education can be defined as “any combination of learning experiences designed with a view to facilitating voluntary actions conducive to health” [13]. Counseling, discussions, individual or group meetings, telephone calls and use of written material for stimulating increased physical activity levels during leisure time or for transportation are actions relating to health education. This type of strategy has as main advantages: a) Encouraging autonomy to the adoption and practice of physical activity; b) Health promotion of a more integrated way with other variables important to public health; c) Empowerment through the development of self-efficacy, social support from the encouragement and incentive to exploit the available environment for physical activity; and d) Being a multicomponent instructional program [14,15]. This type of strategy depends on a well-trained team of professionals in an interdisciplinary way. The traditional strategies of supervised exercise has as main advantage immediate structure available for exercise programs with professional monitoring and specific location, but may have high costs and generate dependency on program continuity to keep individuals physically active [16,17].

The first study comparing a supervised exercise program and a health education program to improve physical activity in adults was published by Dunn et al. [11]. Over a 12-month intervention period, both supervised exercise program and health education actions produced significant increases in daily energy expenditure and physical activity and diminished percentages of body fat.

In 2008, Opdenacker et al. [12] compared a supervised exercise intervention with an intervention based on telephone calls and access to printed materials for stimulating adoption of physically active habits among a sample of elderly people. After 18 months of intervention, the authors found that the two groups presented similar levels of adherence to the programs and similar increases in physical activity levels during leisure time and for transportation. Recently, review studies have indicated that telephone calls, discussion groups on physical activity practices, e-mails, websites and correspondence are useful strategies for increasing physical activity practice in different populations [18-21]. Several viable alternatives for increasing the physical activity levels among different samples of physically inactive subjects have been seen. However, good proportion of these methodologies was tested on individuals who already presented some type of morbidity. Furthermore, intervention studies conducted on samples from populations living in regions of low socioeconomic level in middle-income are scarce. Baker et al. [14] conducted a meta-analysis aiming to verify the effects of community interventions on physical activity levels and found in 25 selected studies that nineteen studies were conducted in developed countries, demonstrating the scarcity studies in middle-income countries like Brazil. Recently, Hoehner et al. [15] conducted a systematic review of intervention studies to promote physical activity in Latin American countries and found only 19 studies that met criteria for inclusion in the analysis. Of these, only school based physical education was classified as evidence-based.

Brazil has the Unified Health System (SUS), a universal public healthcare system with great potential for health promotion strategies [22]. With the physical activity promotion in SUS, mainly after the National Health Promotion Policy, the physical education professionals had their action field enlarged and acquired an important role in the Family Health Strategy. With the Family Health Strategy, they had the potential to serve and promote physical activity of up to 100 million registered users [23]. The Family Health Strategy is an interdisciplinary field based on the community; therefore, the health care is guided by the dimension of family care and occurs through a multidisciplinary team for a population registered, considering and knowing the different contexts in which they live: households, community groups, businesses and others. Therefore, groups with or without risk factors and with different needs are in one common environment [24].

Moreover, these programs work with health promotion in communities with social and environmental characteristics often different, justifying the attempt to test new strategies to adapt to each situation. The lack of detailed descriptions of the procedures used in interventions to promote physical activity and primary care in low socioeconomic status hampers a better understanding of procedures, and also the possibility of replicating the same study design in other samples and regions. Therefore, the aim of this paper was to describe the methodology of two interventions developed in the Brazilian National Health System for promoting physical activity through the Family Health Strategy in a low socioeconomic region.

## Methods

### Type of study

Non-randomized controlled intervention trial. This study is part of a set of interventions called “Ambiente Ativo” (“Active Environment”), which has the aim of testing methodologies for promoting physical activity that can be implemented within the Family Health Strategy (<http://www.each.usp.br/ambienteativo/index.php>).

### Study location

Ermelino Matarazzo is a district in the extreme east region of São Paulo city. The city’s east region is the most populous, with over 30% of the 11 million people living in São Paulo. This region has grown disorderly over the 1960’s and 1970’s decades and today several social problems coexists, such as a high population density and an average Human Development Index (HDI) below the city’s average (east region average HDI = 0.79). Ermelino Matarazzo district has approximately 113,615 inhabitants, a population density of 13,059 inhabitants per km^2^, and 70% of the adults are physically inactive during leisure time. A survey with a representative sample of adults living in Ermelino Matarazzo in 2007 showed that 61.6% classified sidewalks as bad for walking and 66.9% reported that the green areas have bad quality. Most adults reported the presence of waste (60.6%) and open sewers in the streets (71.6%), the lack of crosswalks in the streets (67.5%) and those drivers do not respect the existing crosswalks (83.8%). In addition, 78.5% reported that they feel unsafe to walk in the neighborhood at night [25].

Primary healthcare units are the basic physical structure for attending public healthcare settings users. Ermelino Matarazzo currently has six primary healthcare units, and three of them provide attendance through the Family Health Strategy. Together, these teams provide attendance for approximately 55,000 people, or 50% of the population of this district. The Family Health Strategy is a way of organizing and strengthening primary healthcare, in which reorganization of the healthcare model is sought through expanding access to primary care and qualifying its actions. These primary care actions are centered on the health promotion approach and constructed based on reorientation of healthcare professionals’ practice. Moreover, with the Family Health Strategy, there is the possibility of setting up comprehensive longitudinal care for families. The healthcare professionals involved should establish bonds of trust and responsibility with the individuals, families and communities that they follow up.

### Sample

The inclusion criteria for this study were that the subjects needed to be 18 years of age or older on the date of being approached regarding the interview, needed to be living in the home of the family that was drawn, could not be practicing any form of leisure-time physical activity during the month preceding the interview and could not be practicing physical activity for transportation (walking or cycling) of duration greater than or equal to 150 minutes in the week preceding the interview. The exclusion criteria were as follows: a) type 2 diabetes; b) severe arterial hypertension or using beta-blockers for treating hypertension or cardiovascular disease; c) a health problem or disease that would make the individual incapable of leaving home and making the journey to practice physical activity at the time of the interview; d) diseases at advanced stages, such as cancer, cirrhosis, chronic kidney disease, Chagas disease, chronic obstructive pulmonary disease, chronic bronchitis, osteoporosis or severe depression (information gathered by questionnaires); e) a cognitive problem or disease that would prevent the individual from answering the questionnaire alone; f) morbidly obese, with a body mass index (BMI) greater than or equal to 40 kg.m^-2^; g) plans to move house over the two-year period subsequent to the date of being approached; and h) pregnancy. It was defined that all members of the family drawn who were not covered by any of the exclusion criteria would be invited to participate in the study. According to published data, the adhesion to interventions (subjects who received the invitation and agreed to participate) was 63% [26]. Therefore, the challenge of both interventions was to stimulate a more active lifestyle for individuals who are not initially engaged in leisure-time physical activity, without any kind of chronic disease, and not considering becoming physically active as a priority.

To calculate the sample size, results from previous representative population-based surveys among adults living in Ermelino Matarazzo were used [27]. For adults living in Ermelino Matarazzo who were not active in transportation, the mean time of leisure-time physical activity was 68.1 minutes per week (standard deviation = 146.1 minutes.week^-1^) [28]. For the individuals targeted in this intervention study (adults who were physically inactive during leisure time and insufficiently active in transportation), the goal was to reach a mean 150 minutes of leisure-time or commuting physical activity per week. The goal of stimulating the practice of 150 minutes of physical activity during leisure time or commuting is in agreement with Brazilian studies published previously [29,30]. The main reason for this choice instead of working with the recommendation of 150 minutes of total physical activity is based on that in middle-income countries such as Brazil, the practice of occupational or domestic physical activity has compulsory feature and is little related to pleasure or health promotion, unlike the physical activities in leisure or commuting, made voluntarily. Study involving South Asian-Surinamese, African-Surinamese-Dutch and European adults [31] observed that different socioeconomic levels do not show the same association for physical activity during leisure time or commuting, reinforcing the need to stimulate both types of physical activity when targeting the promotion of health in the population.

The following factors were used to determine the size of the intervention group: standard deviations for the group of adults in Ermelino Matarazzo who were not active in transportation; the goal of mean increase; standardization using a two-tailed test for comparison between means; intraclass correlation coefficient of 0.010 (because of the prior selection of the primary healthcare units from which the subjects were recruited); significance level of 5%; and test power of 80%. This procedure showed that at least 30 individuals would be needed per intervention group. It was considered that the loss would be 25%, and the sample size was corrected thus: n’ = 30 / 0.75 = 40 individuals per group. In all, 157 individuals were selected, and these were divided into three groups: health education (n = 54), supervised exercise (n = 54) and control group (n = 49). Details on the selection process of the sample were published in another paper [26].

### Selection of the primary healthcare units and allocation of the proposed intervention

The primary healthcare units which provided Family Health Strategy attendance were selected. To avoid interference between interventions, it was established that the subjects would only participate in the intervention destined for their primary healthcare units of origin. Individuals attended by the *Unit one* primary healthcare unit received the supervised exercise program, while those attended by the *Unit two* primary healthcare units received the program based on health education. Meanwhile, the individuals attended by the *Unit three* primary healthcare unit were defined as the control group.

Information from the 2007 epidemiological study conducted in Ermelino Matarazzo [28] were used to characterize the region of intervention and assist in the organization and development of intervention strategies. Regarding the level of physical activity, 52.9% of adults were classified as physically active (150 minutes of PA per week), while 16.0% were classified as physically active during leisure time. Regarding the level of education, 60.9% had eight years or less of study and only 10.8% had 12 or more years (at least high school). In addition, the physical activity was associated with greater social support, better perception of security, presence of clubs and low level of pollution in the region studied. This information was of great importance at the time of preparation of materials and training of professionals for intervention, because the low level of education demanded that information passed on to participants should be adequate to their level of understanding. Regarding the environmental characteristics of the region, the absence of any kind of structure to physical activity and proximity to the gymnasium led the researchers to decide that users of unit one could attend only the intervention based on supervised exercise (based on the sports gym of the university). The users of the unit two, being located in a region with presence of clubs, residents' association and public schools, participated in the intervention based on health education. Users of unit three were defined as the control group. The definitions of interventions according to the location of the users also avoided interference between interventions.

### Intervention based on health education

A multidisciplinary team formed by researchers (teachers and graduate students), physical education professionals, a physician, nutritionists and a psychologist drew up several types of approach during the intervention, aiming at working on the participants’ previous experiences, anxieties and availability regarding physical activity practice, along with the degree of access that the environment in which they lived provided them with. This team met every week to discuss the barriers encountered in the intervention and the possible solutions for the problems that occurred during the study period.

This group used different strategies to promote physical activity: a) Group meetings and individual meetings (face-to-face and by telephone) following issues specified in Table 1; b) The community-based, ecologically focused model proposed by Sallis et al. [32], which establishes that physical activity domains are related hierarchically: at the micro level to individual factors (demographic, biological, psychological and family situational factors) and to the perceived environment; and at the macro level to variables of the built environment and policy environment. The aim was to maximize the possibility of engagement in physical activity, through working not only on behavioral change strategies but also on the environment attributes available for physical activity practice. Previous studies demonstrated that the ecological model can be used in physical activity interventions [33,34]. In this intervention, besides discussing intrapersonal barriers, such as motivation, economic barriers to physical activity (first level), were discussed interpersonal barriers with culture of valuing physical activity, social support for physical activity (second level) and strategies to know the structures available for physical activity in the neighborhood (third level).

**Table 1:** Topics at the meetings of the health education group for promoting physical activity

**Topic**	**Objective**
	**1**^**st **^**month**
1- What is physical activity?	To present the multiprofessional team and discuss the concept of physical activity.
2- Physical activity: how much, when and how to do it?	To present different possibilities for practicing physical activity, according to the type, quantity and time of practice.
3- Overcoming barriers to physical activity practice	To discuss the barriers presented by participants and possible strategies for overcoming them.
4- Coping with stress	To conceptualize and present strategies for preventing and coping with stress.
	**2**^**nd **^**month**
5- Physical activity practices	To practice walking and visit public spaces for leisure time in the district.
6- Strategies for a more active day-to-day routine	To present situations and proposals for including habits those are more active in the participants’ routine.
	**3**^**rd **^**month**
7- Surveying dietary needs	To find out about the participants’ dietary experiences and demystify the concept of “diet”.
	**4**^**th **^**month**
8- Aerobic physical activities and cardiorespiratory capacity	To present the concept of aerobic exercises and discuss the recommendations.
	**5**^**th **^**month**
9- Consumption of fruits, vegetables, sugar and salt	To present the healthy diet concept proposed by the Dietary Guide for the Brazilian Population, discuss the fruits and vegetables groups and salt and sugar intake.
	**6**^**th **^**month**
10- Physical activities for strength and flexibility	To present the concept of strength and flexibility exercises and discuss the recommendations.
	**7**^**th **^**month**
11- A healthier day-to-day routine: how to achieve it	To discuss how to adopt healthy practices other than exercise (improved sleep, greater social interaction with family and friends, stimulation of reading and time organization).
	**8**^**th **^**month**
12- Fat, sugar and salt intake and alternative flavorings	To discuss the role of salt and sugar in food flavors; to present herbs and spices as alternative flavorings.
	**9**^**th **^**month**
13- Drawing up a physical exercise session	To conceptualize the following elements of a session: warm-up, peak time and return to calm, through a practical exercise session.
	**10**^**th **^**month**
14- Choosing foods from reading the labels	To practice reading food labels, understand the most important information and seek means for adopting a healthy diet.
	**11**^**th **^**month**
15- Physical activity and nutrition: how to maintain the program	To review the concepts broached and provide strategies for the participants to start or maintain the habits acquired.
	**12**^**th **^**month**
16- Review meeting and conclusion of meetings	To discuss the greatest difficulties and the targets achieved over the period and emphasize the autonomy attained during the intervention period.

The health education program was complemented by actions based on social cognitive theory [35] and self-determination theory [36], aiming changes in self-efficacy and motivation as a result of interaction between environmental factors (spaces to practice, safety, and companionship), personal aspects (previous experiences related to physical activity, physical fitness level, and expectations) and behavioral factors (practice of enjoyable activities and achievement of desired benefits). The program was developed to promote autonomy for physical activity practice using motivation strategies and guidance to overcome barriers based on the interaction of these factors.

Different health-related topics were elaborated for each meeting. The topics defined for the meetings were based on the most important information in the academic literature relating to physical activity and nutrition (Table 1). Most of the content was directed towards physical activity and focused on concepts, recommendations [2], overcoming barriers, health-related physical fitness, recognition of supportive places for physical activity practice, notions of exercise and practical classes on physical activity (total of nine meetings). The intervention also had meetings relating to nutrition that focused on surveying dietary needs, consumption of fruits, vegetables, salt and sugar, choosing healthier foods and understanding labels (total of four meetings). Additionally, there was one specific meeting to discuss the concept of and how to cope with stress, one that joined physical activity and nutrition (about how to maintain the new habits) and a final one to review all contents. During the meetings the topics of healthy habits, avoidance of smoking, moderation of alcohol intake and the importance of sleep and social interaction with family and friends were also emphasized.

With the aim of providing different times and days for attending to the participants, five health education groups with eight to thirteen individuals per group were created. The sixteen meetings were held over a 12-month period and were organized as follows: four weekly meetings in the first month, with the aim of creating better bonding; two fortnightly meetings in the second month and, from the third month onwards, one meeting per month.

All meetings were planned to last 120 minutes, including both the theoretical part and 20–30 minutes of physical activity practice. Printed materials about the meetings were handed out to the participants, together with a list of options for physical activities in public spaces in the district, and specific targets to be achieved between the meetings were created in order to maintain the participants’ motivation.

Participants who were absent received the material in their homes, by post, together with a letter inviting them to the meetings. They also received telephone calls giving them information about the topics at the meetings. All participants received text messages (SMS) to their mobile phones stimulating them to engage in physical activity (“Accumulate at least 30 minutes of physical activity per day: your health will be grateful”).

### Intervention based on supervised exercise program

The second intervention consisted of offering an exercise program in groups supervised by instructors, lasting 12 months. The exercise sessions was held near the residence of the participants (within 0.5 miles), in the sports gym of the university, allowing them to reach the place without using cars or spending time on public transport. The participants were divided into five groups of ten to fifteen individuals each and the training sessions were planned to last 60 minutes. The program that was drawn up followed the recommendations of the American College of Sports Medicine [1] and was based on aerobic exercises (walking and running), resistance exercises and stretching exercises. Throughout the intervention, the frequency of the exercise program was three sessions per week, and the intensity and number of sessions involving aerobic or strength exercises varied according to changes in the training protocol (Table 2). All sessions included a warm-up period and a relaxation period with stretching exercises.

**Table 2:** Planning of the training for the supervised physical exercise group

**Components**	**Description**
	**1^st^ phase**
Aerobic program	Three sessions per week (55%-65% of maximum heart rate), consisting of one session of 40 minutes and two of 20 minutes.
Strength program	Two sessions per week of 10 exercises with free weights and adapted equipment: two sets of 30 seconds for performing each exercise and one minute of interval.
Resistance training exercises	Bench press (chest), bent over barbell row (back), side lateral raise (shoulders), arm curl (biceps), barbell triceps extension (triceps), leg curl (hamstrings), box squat (quadriceps), thigh abductor (thigh), standing calf raises (calves) and crunches (abdominal).
Talks	1- Basic care for practicing exercise; 2- Nutrition.
Extras	1- Capoeira session; 2- Pilates session.
	**2^nd^ phase**
Aerobic program	Three sessions per week (60%-70% of maximum heart rate), consisting of one session of 40 minutes and two of 20 minutes.
Strength program	Two sessions per week of 10 exercises with free weights and adapted equipment: two sets of 15 repetitions of each exercise and one minute of interval.
Resistance training exercises	Bench press (chest), bent over barbell row (back), side lateral raise (shoulders), arm curl (biceps), barbell triceps extension (triceps), leg curl (hamstrings), box squat (quadriceps), thigh abductor (thigh), standing calf raises (calves) and crunches (abdominal).
Extras	1- Gymnastics circuit; 2- Volleyball circuit.
	**3^rd^ phase**
Aerobic program	Alternating between one and two sessions per week (65%-75% of maximum heart rate). Sessions of 40 minutes.
Strength program	Alternating between one and two sessions per week of 10 exercises using professional gym equipment and free weights: two series of 15 repetitions, one minute of interval and fortnightly weight adjustments.
Resistance training exercises	Bench press (chest), bent over barbell row (back), side lateral raise (shoulders), arm curl (biceps), barbell triceps extension (triceps), leg curl (hamstrings), box squat (quadriceps), thigh abductor (thigh), standing calf raises (calves) and crunches (abdominal).
Extras	1- Aerobic gymnastics; 2- Dancing.
	**4^th^ phase**
Aerobic program	Alternating between one and two sessions per week (75%-85% of maximum heart rate). Sessions of 40 minutes.
Strength program	Alternating between one and two sessions per week of 10 exercises using professional gym equipment and free weights: two series of 15 maximum repetitions, one minute of interval and fortnightly weight adjustments.
Resistance training exercises	Bench press (chest), wide-grip lat pull down (back), side lateral raise (shoulders), arm curl (biceps), barbell triceps extension (triceps), leg press 45^o^ (hamstrings), leg extensions (quadriceps/hamstrings), seated leg curl (hamstrings), calf press on the leg press machine (calves) and crunches (abdominal).
Extras	1- “Step” exercise session; 2- Indoor soccer.

The intensity of the aerobic exercise was controlled using heart rate monitors and the Borg scale [37]. The maximum heart rate and the respective training target zones were calculated using the formula proposed by Tanaka et al. [38]. The training load was progressively raised every two months, through increases in the volume and intensity of the aerobic exercises.

Muscle strength was worked on through weight training, consisting of ten exercises performed as a circuit during the first three months of the program, with the number of repetitions limited to an execution time of 30 seconds and with 30 to 60-second intervals between the exercises. From the fourth to sixth month, the program became an alternation of segments containing two sets of 15 repetitions with 60-second intervals. From the sixth month onwards, the intensity was changed to 15 maximum repetitions, while maintaining two sets and a 60-second interval between the exercises and organized in the following order: bench press, half squat, bent over barbell row, leg extensions, side lateral rise, leg curl, arm curl, crunches and barbell triceps extension. In addition, from the sixth month onwards, load adjustments were made at every five sessions. Special sessions of physical activity, such as capoeira, gymnastics, dancing, Pilates, volleyball, indoor soccer and step exercises were held once a month throughout the 12 months of the intervention.

If participants were absent from three consecutive training sessions, the program supervisor would telephone to find out why they were absent, emphasize the importance of attending and, if necessary, suggest a new day and time for the participant to continue in the program. Furthermore, if necessary, the participants could make an appointment with the physician of the research group.

### Control group

The participants in the control group underwent all the procedures relating to the physical evaluation tests and measurements and received all the results in their homes.

The hypothesis for this study is that both interventions are able to modify the physical activity level of the participants, which at the beginning of the study were classified as inactive in leisure and insufficiently active in commuting.

### Assessments

#### Primary outcomes

The weekly physical activity level (last week in relationship with evaluation date) was assessed by the long version of the International Physical Activity Questionnaire (IPAQ) and the 24-hours physical activity recall.

It were used the leisure- and transport-related modules of the long IPAQ, applied in the form of interviews and standardized to assess the physical activity of the last seven days prior to the interview date. In the transport-related module, were investigated walking and bicycle use as modes of transportation, in addition to weekly frequency and daily duration of each type of these activities. In the leisure-time module, were assessed walking, moderate and vigorous physical activity. Were also investigated types of moderate and vigorous activities (up to three moderate and vigorous, separately) and the weekly frequency and daily durations of each activity. Validity indicators for adults living in the region where the study was conducted are previously described in Garcia et al. [25].

The 24-hours physical activity recall is based on logging all the activities performed in the 24 hours prior to the interview. Three days in the week and one day in the weekend were evaluated. After the interview, all activities performed and their respective durations were keyed into specific software, which computes the minutes of light-, moderate- and vigorous-intensity activities, according to the compendium of physical activities of Ainsworth et al. [39] and considering sedentary activities those with 0.9 to 1.5 MET, light-intensity physical activities as 1.6 to 2.9 MET, moderate as 3.0 to 5.9 MET, and vigorous those with ≥6.0 MET. For further details on the validation study and the software, see Ribeiro et al. [40] and Osti et al. [41].

Habitual physical activity was evaluated using the Baecke questionnaire [42]. This questionnaire assesses physical activity in the last 12 months and was answered by all participants. The Baecke questionnaire consists of three non-dimensional scores answered using Likert scales. The scores are: 1) physical activity at work (8 questions); 2) exercise in leisure (4 questions), and 3) leisure- and transport-related physical activity (4 questions). Evidences of instrument validity were previously described in Florindo et al. [42,43] and Garcia et al. [25].

Objective physical activity measurements were made using the Actigraph GT1M and GT3X accelerometers [44] and the Digiwalker CW 700 pedometer [45] over a seven-day period. The pedometers and accelerometers were used in the waist. The accelerometer was attached by a strap and the pedometer was attached directly on garments. The participants were instructed to initiate the use of the devices upon waking and to remove it only for sleeping, bathing or performing underwater activities. Text messages were sent daily to all individuals in order to remind them about the proper use of the devices, considering the usual time of awakening provided by the participants. The data recorded on pedometers and accelerometers were downloaded immediately after the return of both devices. The data were collected by biaxial (GT1M) and triaxial (GT3X) accelerometers, so the cutoff points were calculated based only on the vertical axis through the ActiLife software version 6.8.

#### Secondary outcomes

The secondary outcomes were: body mass, Body Mass Index, body circumferences (upper arm, waist, hip and upper leg) [46], arterial blood pressure, estimated cardiorespiratory fitness at rest (polar fitness test) [47], flexibility (sit and reach test) [48], upper-limb strength (handgrip strength test) [48], abdominal strength (sit-up test) [48], and fasting blood tests (total cholesterol, HDL, LDL, triglycerides, C-reactive protein and fasting glycaemia).

The following variables were also collected, using questionnaires: a) social and demographic variables (age, gender, income, education, marital status); b) barriers to practicing physical activity [49]; c) scale of environmental perceptions for practicing physical activity [27]; d) alcohol consumption and smoking; e) use of medications; f) sleep quality; g) quality of life; h) readiness for exercise practice (PAR-Q) [50]; and i) 24-hours food recall (number of meals and amount of fat, protein, carbohydrate, fruits and vegetables consumed daily).

#### Assessment times

**Figure 1:**
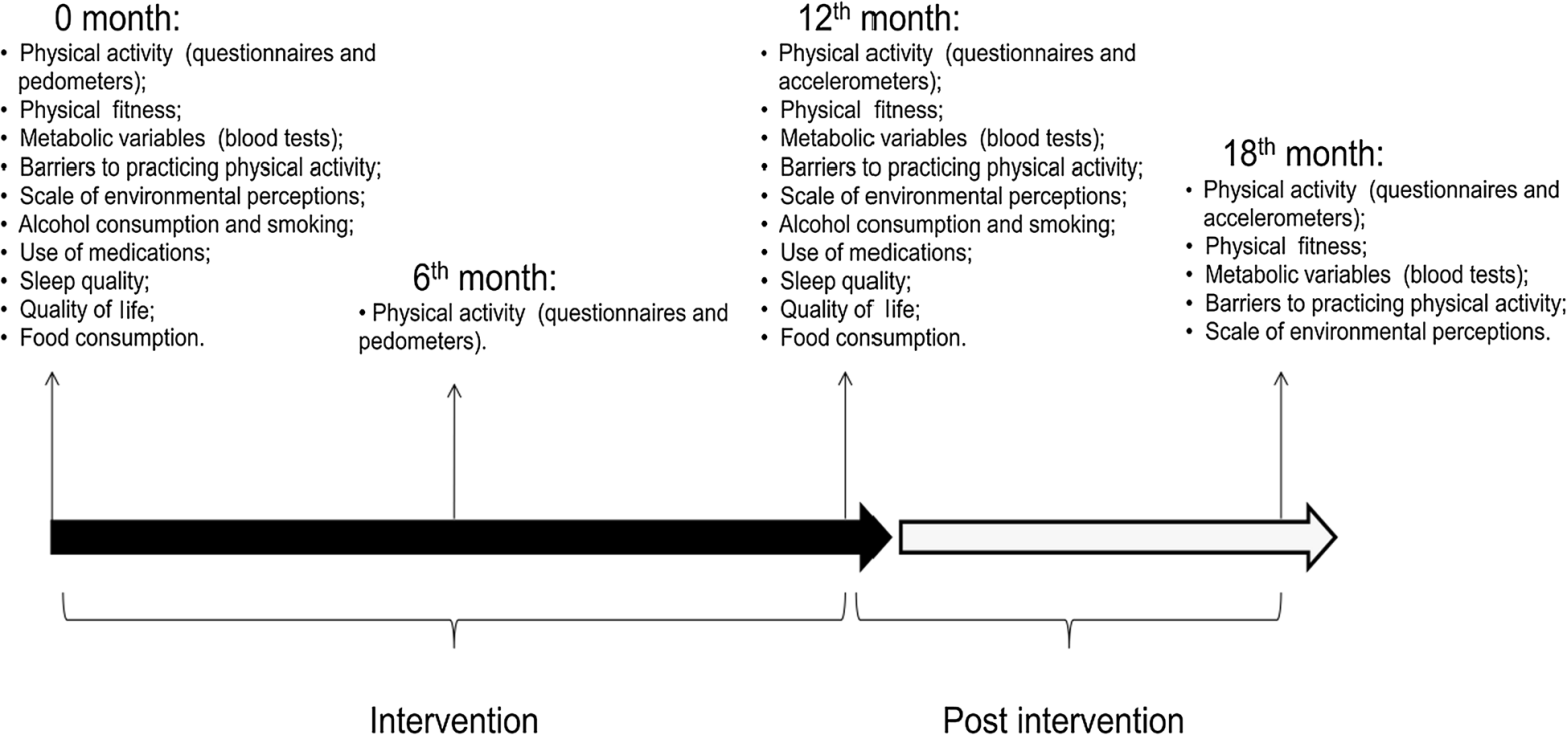
Period of interventions and times of primary and secondary assessments.

This study had four assessment times: just before starting the interventions, after six months of intervention, after 12 months of intervention (end of intervention) and six months after the end of the intervention. Data gathering was done in the same way at all the assessment times, except six months after the beginning of the interventions, when only the primary outcomes were evaluated (Figure 1). Data collection began in February 2011 and the last assessment phase finished in December 2012.

### Ethical issues

This study was approved by the Research Ethics Committee of the Municipal Health Department of São Paulo on September 8, 2010 (protocol number 0072.0.162.000-10) and Research Ethics Committee of the University of São Paulo School of Public Health (protocol number 08437712.6.0000.5421) and was registered in the database of the Brazilian Clinical Trials Register and in the International Clinical Trials Database (identifier: NCT01330836).

## Discussion

This paper presents the methodology of a study that assessed two interventions for promoting physical activity, which were applied within the primary healthcare settings and directed towards adults. The sample for this study was composed of healthy subjects who were physically inactive during their leisure time and insufficiently active in transportation. They were living in a region of low socioeconomic level in Sao Paulo city, Brazil, in an area covered by the primary care model known as the Family Health Strategy. Thus, this intervention was characterized as a primary preventive proposal for healthcare through stimulation of physical activity practice and healthy habits.

The intervention consisting of supervised exercises solely stimulated physical activity in leisure time, exercise and sports through providing a specific location for practice using equipment and open space for walking or running, and sessions involving other forms of physical activity, such as games and dancing. On the other hand, the intervention based on health education promoted physical activity both during leisure time and for transportation, through discussions on how, when, where and how much to practice. Starting from previous experiences that the users brought into the discussions, the activities were directed according to the needs, difficulties and preferences of each group of participants and the availability of public spaces close to their homes. Furthermore, this intervention was aligned with the foundations of health promotion, with discussion on topics relating to diet, stress and other types of health-related behavior. Although this intervention had the main aim of developing empowerment, it did not provide regular exercise sessions, except for a few body experiences that were put forward for educational purposes. Thus, for the participants to change their behavior and become physically active, they needed to develop autonomy and overcome their own barriers against physical activity practice, as well as needing to take advantage of the spaces available in the district where they lived.

Two published studies have used proposals similar to those of the present study in comparing interventions based on structured exercise sessions and health education [11,12]. Dunn et al. 1999 [11] compared two types of intervention for increasing energy expenditure and modifying the level of physical activity. The lifestyle group (n = 121) was advised to start to do 30 minutes of physical activity per day and participated in meetings lasting for one hour per week, for four months, and then fortnightly until completing six months of intervention. After this intensive period, the meetings were held every month for six months, every two months over the next six months and, finally, every three months to complete 24 months of follow-up. Meanwhile, the structured exercise group (n = 114) participated in an aerobic exercise program at intensities of 50 to 80% of the maximum oxygen intake, with weekly frequencies of three to five days per week for six months and then meetings every three months to do group activities. In addition, the participants received materials about the benefits of physical activity, delivered to their homes. However, differing from the present study, that intervention did not have a control group and the subjects were already registered in a larger project (Project Active) [51].

The study of Opdenacker et al. (2008) [12], which followed up 141 elderly subjects over an 18-month period (12 months of intervention and six months of follow-up), had a control group (n = 46), a structured exercise group (n = 49) and a health education group (n = 46). The exercise group participated in 12 months of weight training and aerobic activities in three sessions per week, at intensities ranging from eight to twenty maximum repetitions for the weight training, and 70 to 80% of the reserve heart rate in the aerobic exercises. Participants of the health education group were stimulated to incorporate physical activities into their daily routine. In an individual session, each participant received information on how to do exercises at home, and also received materials containing photographs and instructions for strength, flexibility and aerobic exercises. The information was reinforced through 16 telephone calls (four calls in the first two months and one call per month until the end of the intervention) and group conversation (five meetings per month). In addition, for the health education intervention, the authors used different theoretical models in order to develop autonomy and improve participants’ self-efficacy and motivation to practice physical activity focused on environmental, personal and behavioral factors [32,35,36]. However, the basic methodological difference between the study of Opdenacker et al. and the present study lies in the characteristics of the sample (individuals over the age of 60 years in their study) and the fact that all subjects in their study were volunteers who responded to announcements in newspapers, radio broadcasts and letters.

Both interventions in the present study used telephone calls and printed materials to reinforce, stimulate or restore participation of the subjects in the interventions. In the health education group, the telephone calls were made as a way of transmitting the content to participants who were absent from the meeting. The printed material was handed out at the end of the meeting or sent by post to those who were absent. In the supervised exercise group, the calls were used to ask why participants had been absent and to strengthen and motivate their participation in the exercise program.

Many studies have used telephone calls and printed material as a strategy for increasing the level of physical activity [52-60]. Macfarlane et al. [57] conducted an eight-week intervention involving 50 employees at a university in Hong Kong, who received two telephone calls in conjunction with weekly physical activity sessions. Fontaine et al. carried out a 12-week intervention among 73 individuals with myalgia in the United States who received a telephone call every month in connection with physical activity sessions. Also over a 12-week period, Clark et al. [52] conducted an intervention among 100 users of the British National Health Service, who received three telephone calls over this period, with the aim of stimulating physical activity practice and healthy habits. Wilcox et al. [60] devised an intervention that solely involved telephone calls among 118 elderly women for three months. Other papers have presented intervention periods close to what was used in the present study. McMurdo et al. [58] conducted a six-month intervention involving 204 elderly Scottish women who received telephone calls as a way of stimulating and educating about physical activity, along with regular use of a pedometer and educative sessions on physical activity. In the study by Dubbert et al. [54], 181 elderly individuals in the United States only received telephone calls as the means of intervention (20 calls over a 10-month period). Kerr et al. [56] conducted a 12-month intervention involving 411 overweight and obese subjects in the United States, with telephone calls every three months, in conjunction with internet access made available to the subjects for them to obtain information on the benefits of physical activity. Among a sample of 434 diabetics who were users of the Australian public healthcare system, Reeves et al. [59] organized an intervention lasting 12 months that involved ten telephone calls over the first four months and use of a pedometer during the remainder of the study.

Studies evaluating interventions for promoting physical activity that are applied to the public system are very important in middle-income countries like Brazil, that have a universal system and the Family Health Strategy. The Brazilian Ministry of Health has been investing in promoting physical activity over the last seven years. According to data published by Knuth et al. [61], 469 projects for promoting physical activity were in operation in Brazil in 2008, throughout the country, of which 60% were in cities with populations lower than 30,000 inhabitants. Their characteristics included use of different spaces for practice (sports courts, cycle ways, healthcare units, walking trails and schools) and different healthcare professionals (physicians, physical education professionals, nutritionists, psychologists, community health workers), and the proposals differed regarding the lifestyle changes targeted (improvement of diet, combating smoking and crime prevention, among others). One of these programs is “Academia da Cidade” [62]. It was started in the city of Recife in 2002, with the aim of promoting physical activity among the population and stimulating healthy dietary habits by physical activity programs supervised by physical education professionals, in centers scattered around the whole city, where people can practice aerobic and muscle strength activities at a frequency of three times a week. The “Academia da Cidade” program was evaluated in 2008 and it presented evidence of effectiveness for promoting leisure-time physical activity and the Brazilian government expanded the program, terming as “Academia da Saúde” for health promotion, in addition to promoting physical activity in leisure, encourages healthy eating, enhancement of local culture, especially in areas of higher social vulnerability. Currently, 1,828 Brazilian cities have funding for conducting some type of promotion of physical activity [63]. However, for many of these strategies, no evaluations on their effectiveness have yet been done. Even for the “Academia da Cidade” program, which was a major advance with regard to promoting physical activity in Brazil, there is no randomized and controlled evidence to ascertain the impact of the program on physical activity levels. Furthermore, none of these studies aimed to evaluate the impact of different programs among adults enrolled in the primary care system in order to identify which strategies could be easily implanted by the health professionals as a primary preventive action.

One of the strengths of this study was to adapt the study objectives with the needs of the region. Both interventions were developed after an epidemiological study conducted in the same region during the year 2007, in order to assess the level of physical activity and perceived and objective environment. These results demonstrated that the majority of the adults (70%) living in the region were inactivity in leisure time and served as references to characterize the sample who participated in the intervention. After this step, the researchers contacted the professionals working in the primary health care in the region to prepare interventions. Thus, the experience of primary care professionals working in the area had served as a benchmark to assess the feasibility and effectiveness of interventions designed for this region of low socioeconomic level.

Another important feature was the theoretical basis used in this intervention. The ecological model of physical activity promotion proposed by Sallis [32] was chosen considering the different types of stimulated physical activity (including leisure and transport) and the different levels that influence the practice on these domains (politic, demographic, biological, psychological and family situational factors). However, it is extremely difficult to work with an intervention at all levels of the model, so the researchers decided to focus the intervention in the first two (individual and environmental).

The length of the intervention in the methodological proposals of the present study (12 months) had the aim not only of promoting an initial contact with exercise practice, but also of allowing individuals to make positive changes to their behavior, thereby acquiring new and healthy habits over the medium and long terms that might be maintained. Studies mentioned in the systematic review of Hoehner et al. [15] and Baker et al. [14] showed that long-term studies (at least six months) have a higher chance of achieving important results in physical activity level.

## Results and conclusions

This study described two different proposals for promoting physical activity that were applied to adults attended through the public healthcare system who were living in a region of low socioeconomic level, while respecting the characteristics and organization of the system and its professionals, and also adapting the interventions to the realities of the individuals attended. Results from this study are going to identify which strategy could be more effective to promote physical activity among health and physically inactive adults and could be implanted in the Brazilian healthcare system to control and prevent the non-communicable diseases burden.

## Competing interests

The authors declare that they have no competing interests.

## Authors’ contributions

EPS and AAF established the research, planned interventions, and wrote the article. EHR, LMTG, and DRA assisted in the preparation of the health education intervention and writing the manuscript. VVG and MSA assisted in the preparation of the intervention based on supervised exercise and writing the article. All authors read and approved the final manuscript.
